# Supplementary material for: Single-cell transcriptomes identify patient-tailored therapies for selective co-inhibition of cancer clones
Source: Nat Commun. 2024 Oct 3;15:8579. doi: 10.1038/s41467-024-52980-5 (PMC11450203; doi:10.1038/s41467-024-52980-5)
Supplement: Supplementary file 1 — Supplementary Information [file 41467_2024_52980_MOESM1_ESM.pdf]

Supplementary Information for

Single-cell transcriptomes identify patient-tailored therapies for selective co-inhibition of cancer clones

Aleksandr Ianevski, Kristen Nader, Kyriaki Driva, Wojciech Senkowski, Daria Bulanova, Lidia Moyano-Galceran, Tanja Ruokoranta, Heikki Kuusanmäki, Nemo Ikonen, Philipp Sergeev, Markus Vähä-Koskela, Anil K. Giri, Anna Vähärautio, Mika Kontro, Kimmo Porkka, Esa Pitkänen, Caroline A. Heckman, Krister Wennerberg, Tero Aittokallio

Supplementary Information

- **Supplementary Tables 1-4** Pages 1-4
- **Supplementary Figures 1-11** Pages 5-14
- **Supplementary References** Pages 14-16

Supplementary Table 1 | AML patient samples used in the study.

Pt	Disease stage	scRNA-based blast (%)	Clinical morphological blast (%)	FAB type	ELN22 risk class	Potential driver mutations	Chromosomal abnormalities	No. cells after QC[a]
1	Diagnosis	79.3	80	NA	Favorable	NPM1, DNMT3A, NRAS, IDH1, EML4	Normal karyotype	2483
2	Diagnosis[b]	38.1	70	M1	Favorable	NPM1, TET2, USP8	Normal karyotype	2874
3	Refractory[b]	56.7	42	M1	Favorable	NPM1, TET2, HDAC 1,2,7	Normal karyotype	2421
4	Diagnosis	80.3	91	M1	Favorable	RANBP2, NPM1, IDH1, FLT3	Normal karyotype	2140
**5**	Relapse	54.4	26	M5	Intermediate	LZTR1, AR, ICOSLG	Normal karyotype	2365
**6**	Relapse	40	14	M1	Intermediate	FLT3, PTPN11, TP53BP1	Polyploidy	5677
7	Diagnosis	40.8	36	NA	Adverse	RUNX1, BCORL1, PTPN11	Monosomy 7 + intra-chromosomal translocation Chrom 3	9340
8	Refractory	22.8	40	NA	Adverse	DNMT3A, ERG, U2AF1, BCOR	Normal karyotype	4461
9	Diagnosis	50	70	M1	Adverse	BCOR, IDH2	Normal karyotype	3921
10	Diagnosis	65.5	65	M2	Adverse	WT1, CCND2, CEBPA	del(5q)	3111
**11**	Diagnosis	42.1	32	M2	Intermediate	VAV1	Normal karyotype	5697

**12**	Refractory	65.3	65	M2	Adverse	MN1, MAP2K2, ETV6, FOXP1	del(5q)	3610

Age range: 21-75 years; female/male ratio: 7/5.
[a]Quality control: filtering out low-quality cells (see Step 2 in the Methods section).
[b]Paired samples from the same patient at disease diagnosis and refractory stages.
Boldfacing indicates patient samples with enough cells for experimental validation.

Supplementary Table 2 | Clinical and predicted treatment responses for patients with AML.

Pt	Disease stage	Treatment before sampling (response)	Treatment after sampling (response)	Predicted response to treatment after sampling
1	Diagnosis	-	Hydroxyurea (no)	-
2	Diagnosis	-	Azacitidine (no)	-
3	Refractory	Azacitidine (no)<br>Venetoclax (no)	-	-
4	Diagnosis	-	Hydroxyurea (yes),<br>Cytarabine/Idarubicin (yes),<br>Cytarabine/Daunorubicin (yes),<br>busulfan/cyclophosphamide (yes)	Daunorubicin (HM)
5	Relapse	Hydroxyurea (yes),<br>Cytarabine (yes),<br>Cytarabine/Idarubicin (yes),<br>Research drug treatment (no)	Investigational immunotherapy	-
6	Relapse	Cytarabine/Idarubicin (yes),<br>Mitoxantrone/Cytarabine (yes),<br>Amsacrine/Cytarabine/Etoposide (yes),<br>Idarubicin/Cytarabine/Etoposide (yes),<br>Clofarabine/Cytarabine/Etoposide (yes),<br>Azacitidine (no),<br>Azacitidine/Lenalidomide (no)	Mitoxantrone/Etoposide/Cytarabine (yes),<br>Clofarabine/Cytarabine (no)	Mitoxantrone (HM),<br>Etoposide (M)
7	Diagnosis	-	Azacitidine (no)	-
8	Refractory	Cytarabine/Idarubicin (no)	Cytarabine/Mitoxantrone (no),<br>Azacitidine (no)	Mitoxantrone (HM),<br>cyt:clofarabine (M-LC)
9	Diagnosis	-	Cytarabine/Idarubicin/GF (yes),<br>Cytarabine/Mitoxantrone (yes),<br>Cytarabine (yes)	-
10	Diagnosis	-	Cytarabine/Idarubicin (yes)<br>Cytarabine/Idarubicin/Lenalidomide,<br>Cytarabine/Daunorubicin (yes),<br>Etoposide/Mitoxantrone (yes),<br>Lenalidomide (yes)	Etoposide (M),<br>Lenalidomide (M-LC)
11	Diagnosis	-	NA	-
12	Refractory	NA	NA	-

The columns include treatments before and after taking the sample, as well as the clinical response (yes/no), based on the percentage of blasts in the bone marrow of the patient. Predicted response with scTherapy to the treatments after sampling or drugs with similar mechanisms of action. Drug names in red font represent those that were

predicted by scTherapy as effective but filtered out due to low confidence of the predictions. Response prediction: HM, High-to-Moderate; M, Moderate; LC, Low confidence. - scTherapy prediction not possible for the non-targeted patient treatment or clinical response data not available (NA) at the time of the study from unpublished clinical trials.

Supplementary Table 3 | HGSC patient samples used in the study.

Patient ID	Treatment	Stage[a] (FIGO)	Anatomical location of scRNA-seq	Cell types	No. cells before QC	No. cells after QC
Patient 1	Treatment naïve	IIIC	Omentum	PAX8+	1921	1743
Patient 2	Treatment naïve	IVB	Ascites	PAX8+<br>PAX8-	2954	1483
Patient 3	Treatment naïve	IVA	Ascites	PAX8+<br>PAX8-	2178	1934
Patient 4	Treatment naïve	IIIC	Peritoneum	PAX8-	711	564
Patient 5	Treatment naïve	IIIC	Ascites	PAX8-	3666	2399

[a]Stage IIIC (FIGO staging): The cancer is in one or both ovaries or fallopian tubes, or there is primary peritoneal cancer, and it has spread or grown into organs outside the pelvis. The deposits of cancer are larger than 2 cm (about 3/4 inch) across and may be on the outside (the capsule) of the liver or spleen (T3c). Stage IVA: Cancer cells are found in the fluid around the lungs with no other areas of cancer spread such as the liver, spleen, intestine, or lymph nodes outside the abdomen (M1a). Stage IVB: The cancer has spread to the inside of the spleen or liver, to lymph nodes other than the retroperitoneal lymph nodes, and/or to other organs or tissues outside the peritoneal cavity such as the lungs and bones (M1b).

Supplementary Table 4 | Clinical development stages of scTherapy-predicted treatments across 5 cancer types (http://clinicaltrials.gov/).

Indication	Treatment	Phase	NCT identifier number
Breast	abemaciclib	3	NCT05952557,NCT03155997[1]
	alpelisib	3	NCT05501886,NCT04251533
	epirubicin	**4**	NCT01642771[2],NCT01216111[3],NCT00630032,NCT00540800[4],**NCT01199432**,NCT00689156[5],NCT03498716 NCT04301739,NCT03036488,**NCT04136782**,NCT05862064,NCT02455141,NCT04031703,NCT03876886 NCT04296175,NCT01378533,NCT04335669,NCT06112379,NCT00912444
	5-fluorouracil	3	NCT00121992[6],NCT04031703
Lung	etoposide	3	NCT00003364,NCT00717938[7],NCT00632853[8],NCT00002858,NCT00003696,NCT00003606,NCT00003299,NCT00061919,NCT00011921,NCT00433498,NCT00045162,NCT02875457,NCT00002822,NCT00812266
	paclitaxel	3	NCT00003696,NCT00003299[9],NCT00011921,NCT00003317[10],NCT00003589[11],NCT02477826[12],NCT00054184, NCT00795340,NCT00054197,NCT00006049,NCT00054210,NCT00551733[13]
	epirubicin	3	NCT00003606[14],NCT00011921

	docetaxel	3	NCT00074204,NCT00022022[15],NCT00883675[16],NCT00054184,NCT02076477
	tivantinib	3	NCT01244191[17]
AML	daunorubicin	3	NCT00428558[18],NCT00589082[19],NCT04174612,NCT00266136[20],NCT00931138[21],NCT00715637[22],NCT03897127,NCT02013648,NCT00703820,NCT04293562,NCT00927498[23],NCT00363025[24]
	decitabine	3	NCT03941964,NCT05177731,NCT02172872[25],NCT05586074,NCT02348489,NCT01633099,NCT02785900
	etoposide	3	NCT00052299[26],NCT02421939,NCT03504410,NCT03182244,NCT00703820,NCT04293562
	mitoxantrone	**4**	NCT01828489,NCT00052299[26],NCT02421939[27],NCT03504410,NCT03182244,NCT02461537,NCT04293562,**NCT00180102**
	panobinostat	3	NCT04326764
	tipifarnib	3	NCT00093990[28]
HGSC	alpelisib	3	NCT04729387[29].
	cyclophosphamide	3	NCT00003214[30],NCT00004921[31],NCT00002477,NCT04520074,NCT00002819,NCT00068601[32]
	etoposide	3	NCT04000295,NCT04520074
	olaparib	**4**	NCT03737643,NCT02392676,**NCT02476968**,NCT03402841[33],NCT05255471[34],NCT03106987,NCT01874353[33],NCT04729387[29],NCT03534453[35],NCT01844986[36],NCT02282020[37],NCT04884360,NCT04330040,NCT02477644[38],NCT03740165
	paclitaxel	**4**	NCT03737643,NCT05371301,NCT00326456[39],NCT04729608,NCT00657878,NCT00660842[40],NCT00003214[30],NCT03940196,NCT00004921[31],NCT04000295,NCT03398655,NCT04337632,NCT02718417,NCT01239732,NCT01802749,**NCT01706120**,NCT04729387,NCT00002894[41],NCT00003644[42],NCT01684878,NCT03690739,NCT05145218,NCT00002717[43],NCT06072781,NCT00002819,NCT05009082,NCT00189553,NCT00003322[44],NCT00003998[45],NCT03806049,NCT01654146,NCT02470585[46],NCT05281471,NCT03740165,NCT03794778,NCT00006454[47],NCT00028743[48],NCT00483782[49],NCT02631876,NCT00002568[50],NCT05601700,NCT04908787,NCT00189371
Pancreas	5-fluorouracil	3	NCT00417209,NCT00602745,NCT05314998
	paclitaxel	**4**	NCT03721744,**NCT04217096**,NCT01836432[51],NCT03941093,NCT02506842,NCT04229004,NCT05178628,NCT04617821,**NCT05035147**,NCT05751850,NCT04835064,NCT05653453,NCT04674956,NCT06017284,NCT04329949,NCT04935359,NCT02101021,NCT03943667[52],NCT02715804,**NCT04480268**[53],NCT02993731,**NCT03401827**[54]

Boldfaced NCT IDs represent clinical trials that have reached phase 4.

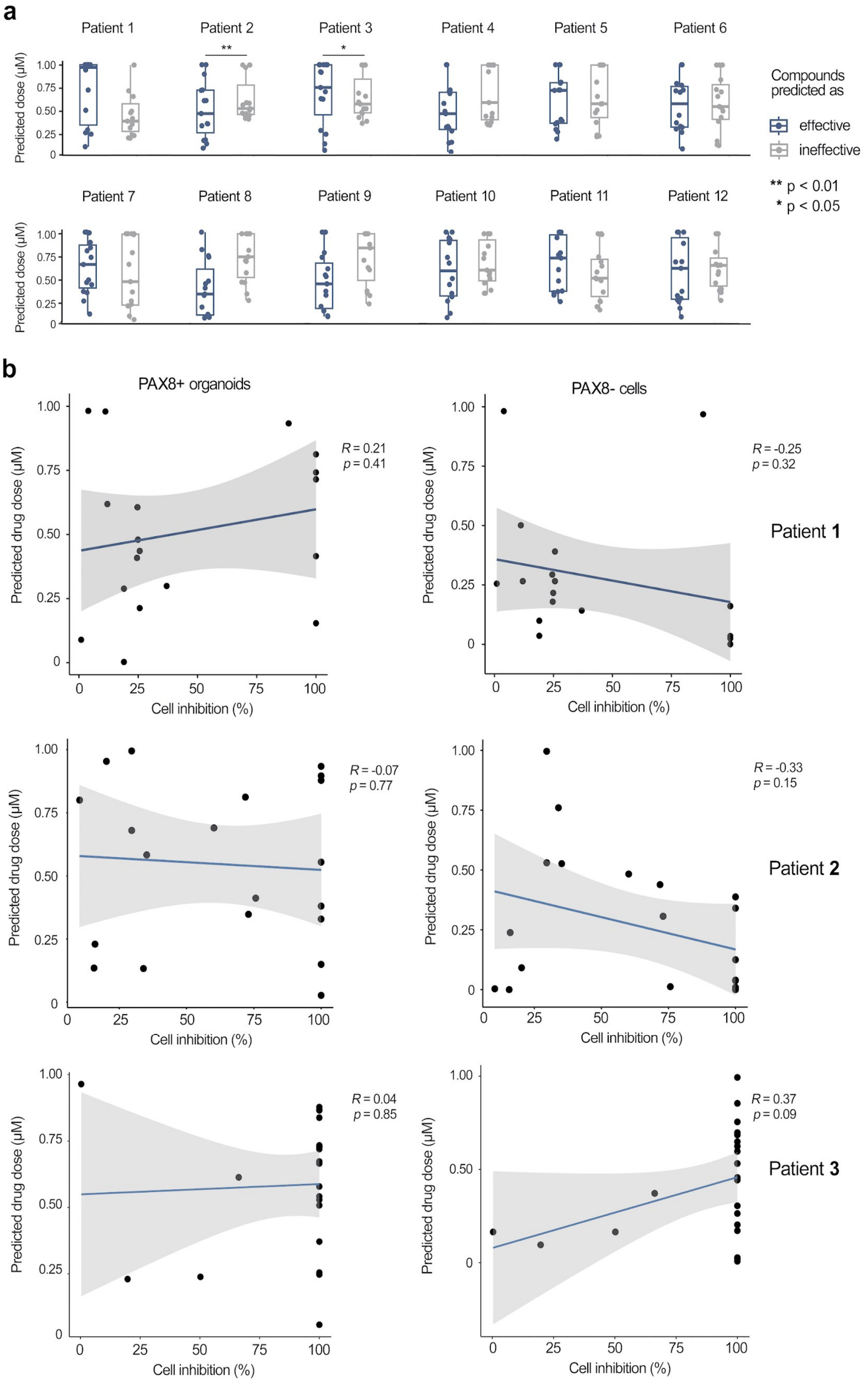

Supplementary Fig. 1 | Drug doses of the predicted single-agent treatments. (**a**) Drug doses (μM) for single-agent treatments predicted by the model to be either effective or ineffective for 12 AML patients (two-sided Wilcoxon test). In general, there is no significant difference in the doses between drugs predicted to be effective or ineffective. Box plots show the median (central line), 25th and 75th percentiles (box edges), and the range within 1.5 times the interquartile range from the box (whiskers). Source data are provided in Source Data file. (**b**) Correlation between the predicted drug doses and the measured PAX8+ (left) or PAX8- (right) cell inhibition across the 18 predicted treatments in the HGSC patient samples. Non-significant correlation based on the correlation test in every patient.

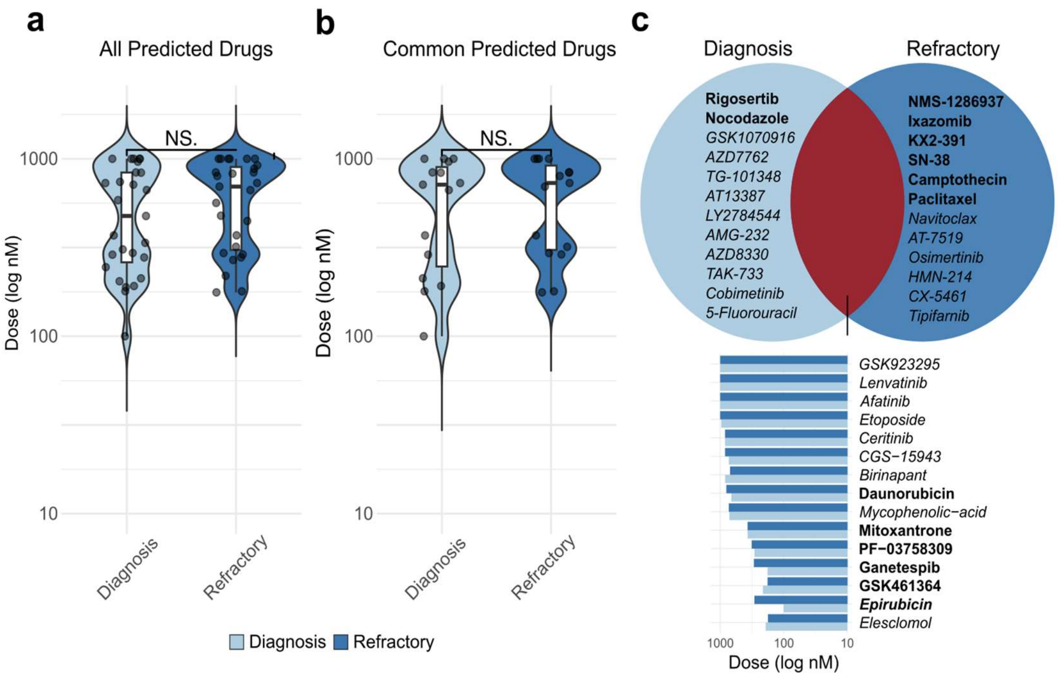

Supplementary Fig. 2 | Predicted effective drugs and doses for the diagnosis and refractory samples of the same AML patient. No significant (NS) differences were found in the predicted effective doses between the paired AML patient samples 2 and 3 from the same individual. (**a**) Predicted doses of all predicted drugs, difference assessed with two-sample Wilcoxon test. Box plots show the median (central line), 25th and 75th percentiles (box edges), and the range within 1.5 times the interquartile range from the box (whiskers). (**b**) Predicted doses of common drugs predicted for both the diagnostic and refractory samples, difference assessed with paired signed rank test. (**c**) Predicted effective drugs in diagnosis and refractory samples of the same individual. scTherapy predicted a total of 27 monotherapies both in the diagnosis and refractory samples, out of which 15 are common between the two samples. Drugs in boldface are those predicted to elicit a high-to-moderate response, while those in italics are expected to produce a moderate response. Epirubicin was predicted in diagnosis and refractory samples with moderate and high-to-moderate responses, respectively. Source data are provided in Source Data file.

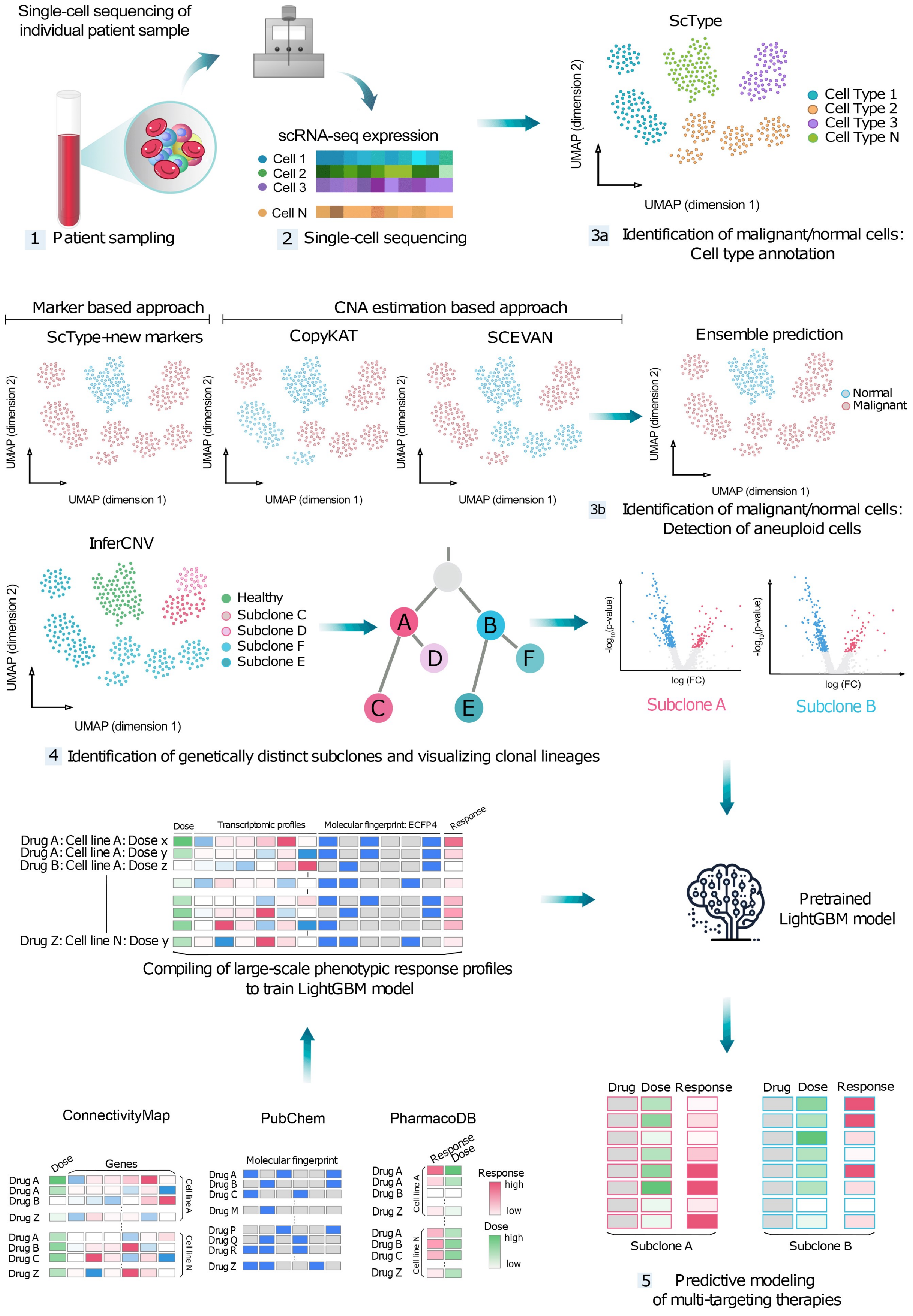

Supplementary Fig. 3 | Schematic workflow of the experimental-computational approach to predicting multi-targeting treatments for an individual AML patient/sample. See Online Methods for details of steps 1-5.

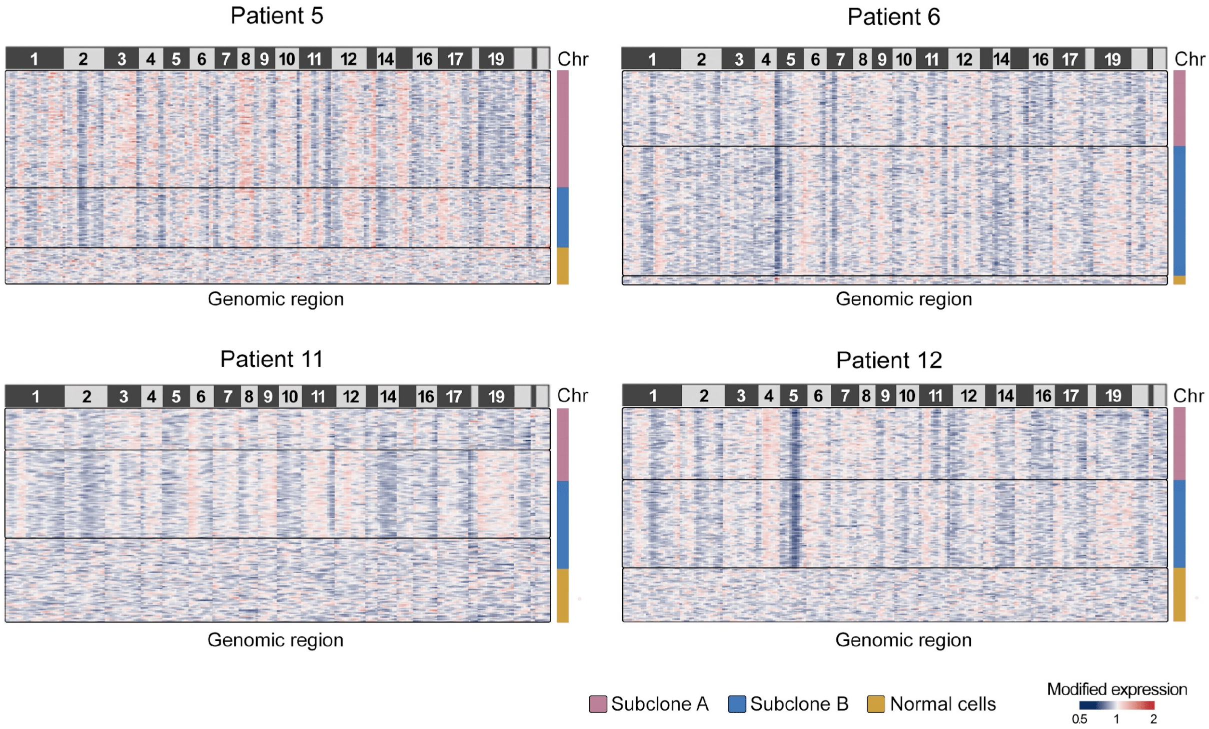

Supplementary Fig. 4 | InferCNV copy number variation analysis of malignant cells from the four AML samples. The heatmaps show a graphical representation of the CNV across the genomic regions, denoted as chromosomes (Chr) at the top of each heatmap. Shades of blue indicate lower levels of modified gene expression, suggesting genomic loss, while shades of red indicate higher levels of modified gene expression, indicative of genomic gain.

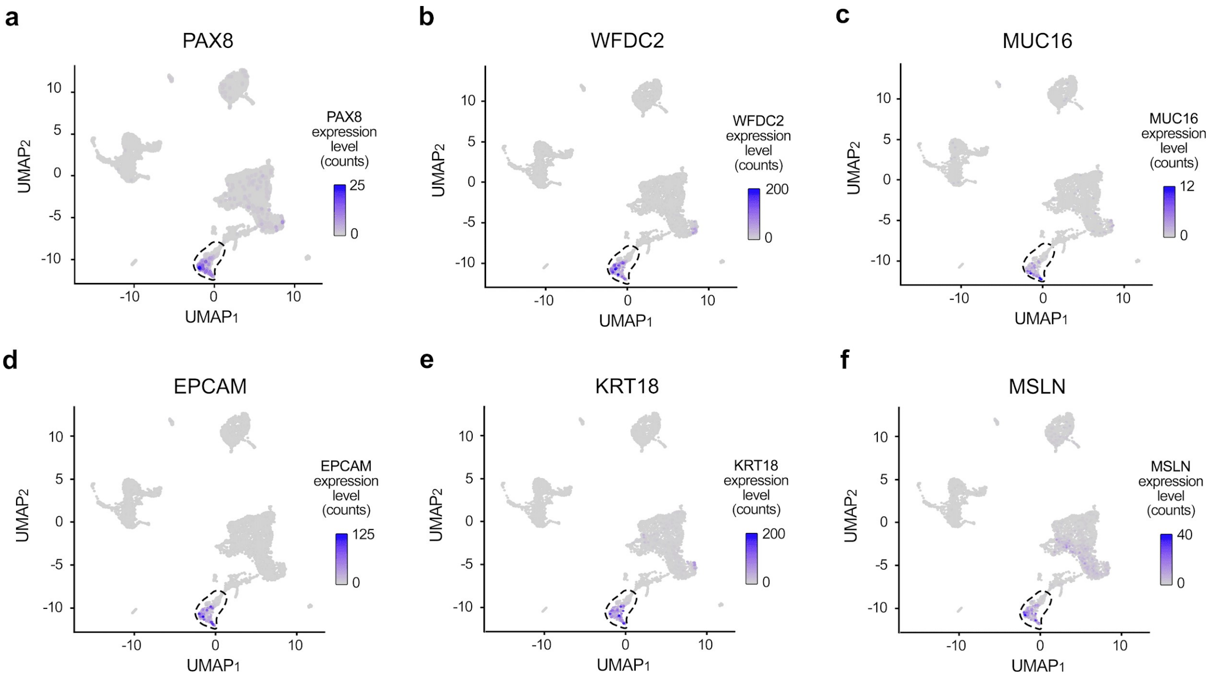

Supplementary Fig. 5 | Expression of the PAX8+ marker genes for the detection of ovarian cancer cell populations. (**a**) PAX8, (**b**) WFDC2, (**c**) MUC16, (**d**) EPCAM, (**e**) KRT18, and (**f**) MSLN. These markers were utilized for the detection of cancer cell populations in the HGSC Patient 1 (highlighted with dashed circles). The number of cells analyzed in Patient 1 as shown on UMAP plots is 4,706 cells.

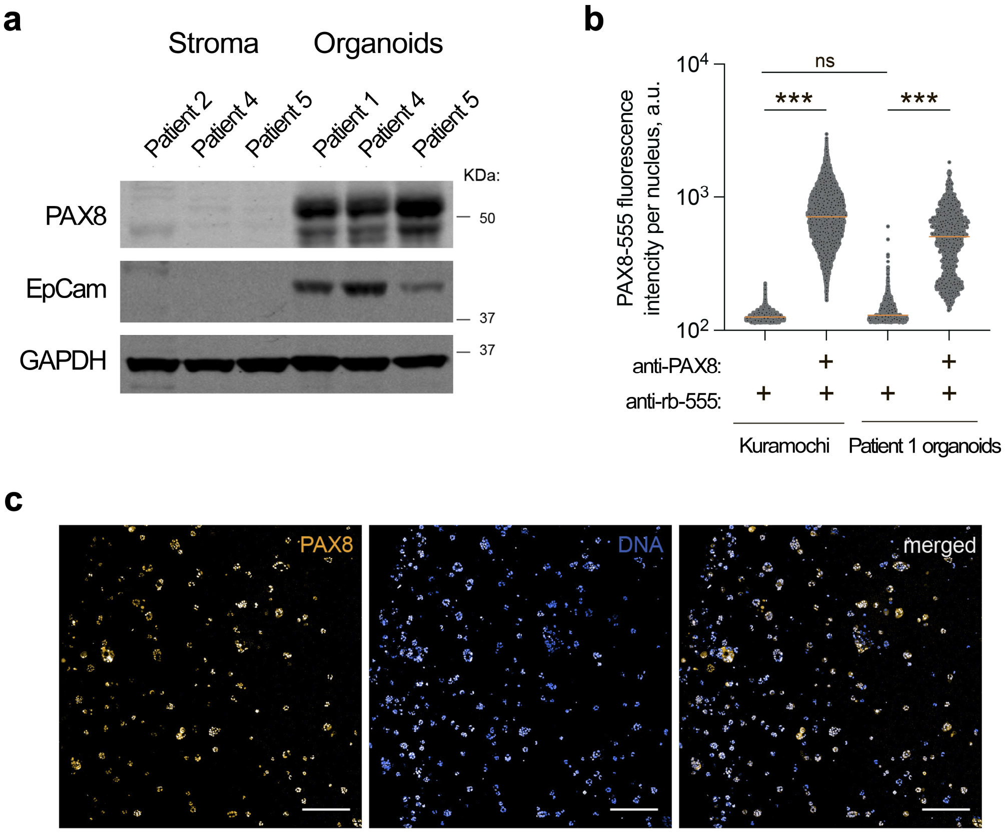

d

Statistical parameter	Kuramochi (control)		Organoids (patient 1)	
	only 2ry-555	anti-PAX8+2ry-555	only 2ry-555	anti-PAX8+2ry-555
N of nuclei scored	13721	13604	2040	1784
Minimum	113,5	167,3	114,2	140,7
Median	125,6	712,4	129,6	503,5
Maximum	225,9	2986	598,3	1832
10% Percentile	117	386	117,8	206,3
90% Percentile	141,4	1273	169,9	883,7
Mean	127,7	782,3	138,9	523,1
Std. Deviation	10,14	362,2	28,63	267,3
Std. Error of Mean	0,0866	3,105	0,6339	6,328
Coefficient of variation	7,941%	46,30%	20,61%	51,10%
**% of PAX8+**	**1 %**	**100 %**	**1 %**	**84 %**

Supplementary Fig. 6 | Experimental analyses in the HGSC patient samples and organoids. (**a**) Immunoblotting for PAX8 and Epcam proteins in the stroma-derived and organoid samples from the representative individual HGSC patients (one experiment). The uncropped images with molecular weight markers are provided in the Source Data file. (**b**) Quantification of the imaging data for PAX8 nuclear expression in Patient 1-derived HGSC organoids (related to **Fig. 3e**), and in one control HGSC cell line, Kuramochi. Statistical significance was determined using a two-sided Wilcoxon signed-rank test; *** $p < 0.001$. Anti-rb-555, secondary goat-anti-rabbit-Alexa555 antibody conjugates. (**c**) Representative images of the immunofluorescence imaging of the nuclear PAX8 expression for the Patient 1 organoids. Scale bar equals to 250 µm. (**d**) Statistical analysis of the imaging data presented in panels b and c. The fraction of PAX8+ objects correspond to the nuclei with PAX8-555 signal higher than mean+3SD for the respective control without PAX8 primary antibodies.

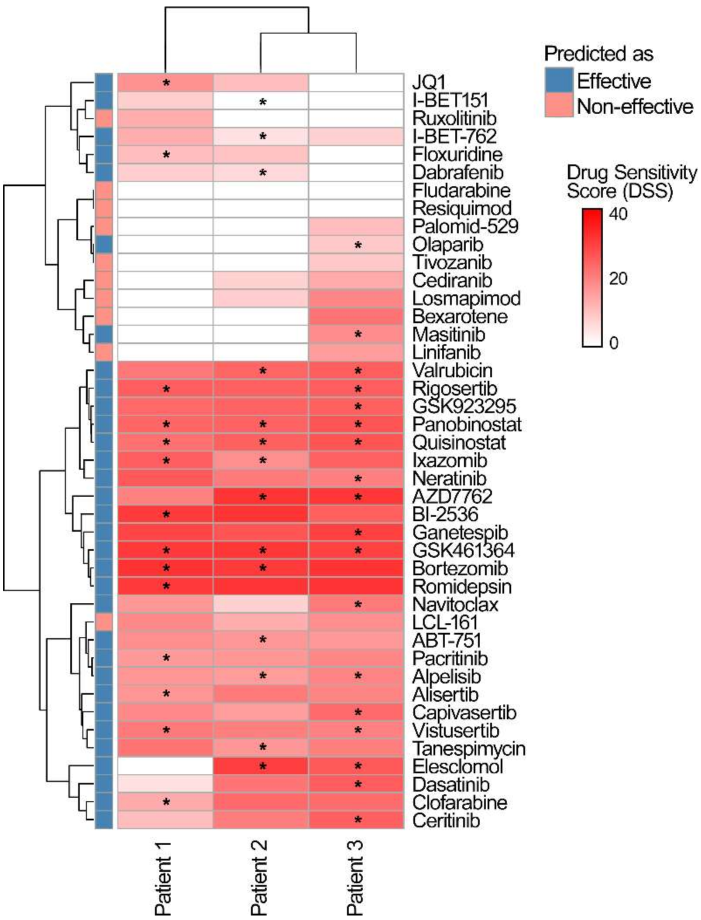

Suppl. Fig. 7 | Heatmap of measured drug sensitivities for treatments predicted either as effective or ineffective in ovarian cancer patients. Blue annotation in the left-hand column indicates that the drug was predicted to be effective in one or multiple ovarian cancer patients. Red color indicates those drugs predicted as non-effective in all the patient samples. *Drug treatments for which high efficacy class was predicted by the scTherapy model, showcasing the accuracy of our predictive model across the three patient-derived organoid lines. Source data are provided as a Source Data file. Scale bar represents Drug Sensitivity Score (DSS) values from 0 to 40 (white to red).

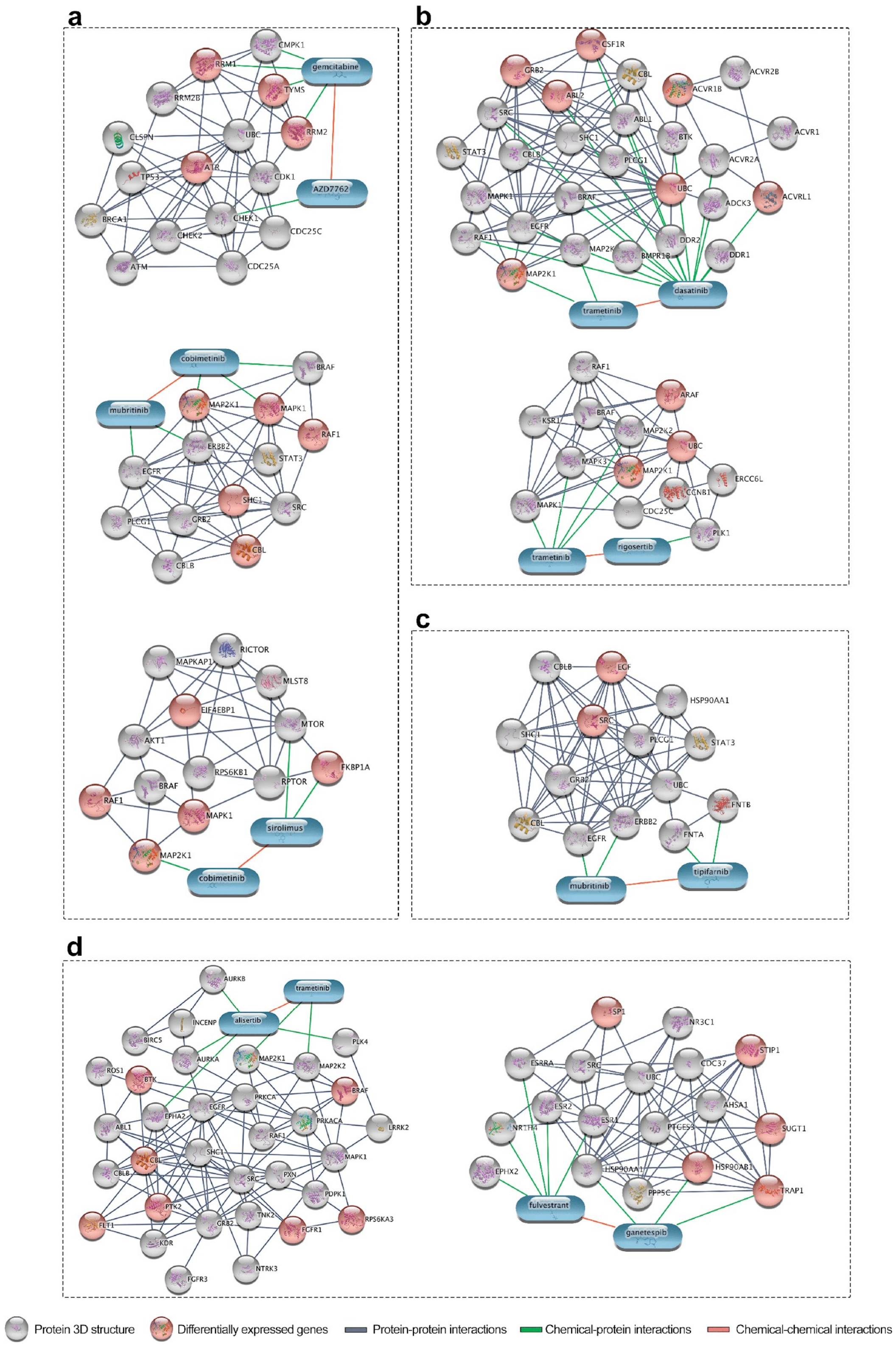

Supplementary Fig. 8 | Interaction networks for the predicted and experimentally validated AML patient-specific combinations. Representative drug combinations, where multiple drug targets were identified as differentially expressed genes between normal and malignant cells for the 4 AML patients: (**a**) Patient 5, (**b**) Patient 6, (**c**) Patient 11, and (**d**) Patient 12. The protein nodes in the networks include the nominal and potent off-targets of the compounds in the combinations, along with differentially expressed genes in the target pathways that may partly explain the observed combination effects in the particular patient cases. The network visualizations were done using the STITCH web-tool (Szklarczyk, D. et al. STITCH 5: augmenting protein-chemical interaction networks with tissue and affinity data. Nucleic Acids Res. 44, D380-384 (2016).

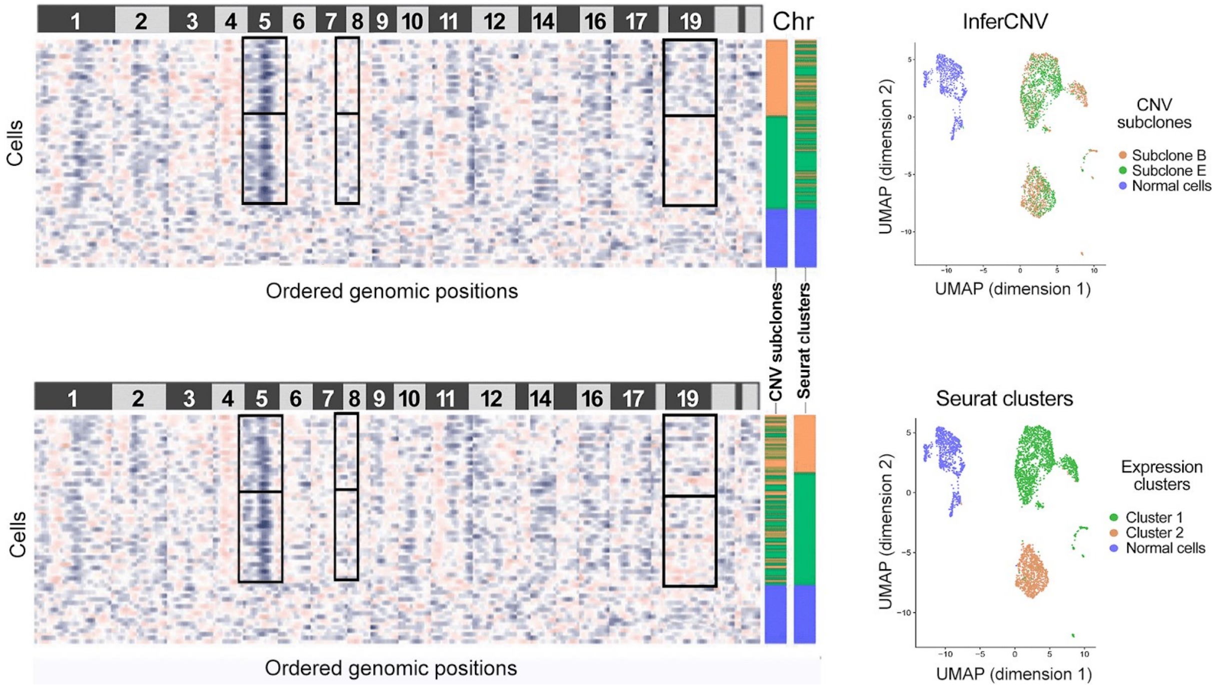

Supplementary Fig. 9 | Genetic clone detection vs. cell clustering. Comparison of cancer subclone characterization using inferred Copy Number Variation (CNV) profiles (upper panel) versus expression-based cell clusters (lower panel) in an AML patient sample 12. Bold rectangles highlight areas with pronounced expression differences, underscoring the effectiveness of CNV-based analysis in uncovering genetic subclonal diversity essential for designing clone-specific treatment options. The number of cells analyzed in Patient 12 as shown on UMAP plots is 3,610 cells.

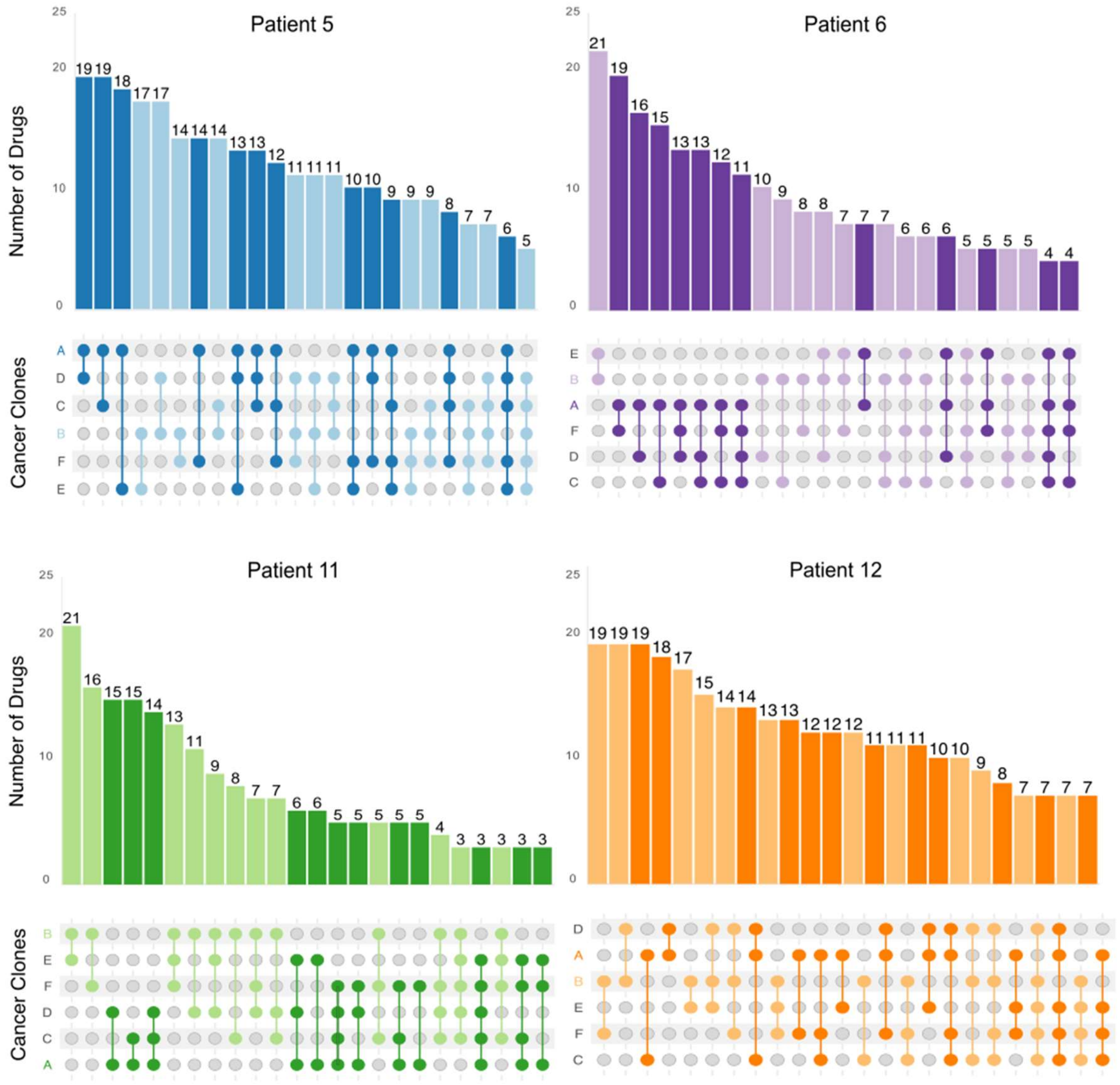

Supplementary Fig. 10 | Overlap in scTherapy treatment predictions among the major and minor subclones across four AML patient samples. The darker bars highlight drug prediction overlaps with the first major subclone, and the lighter bars with the another. The colored subclone labels denote the major subclones, while those in gray denoted minor subclones. The four patient samples correspond to those used in the experimental validation. Source data are provided as a Source Data file.

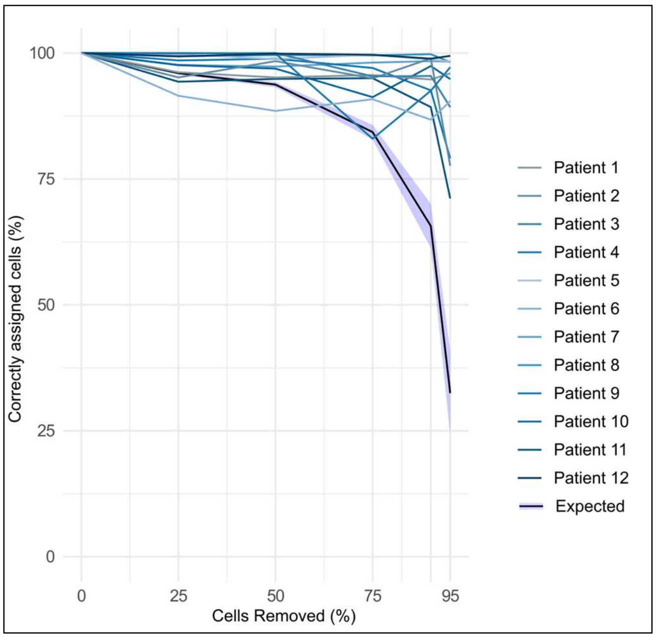

Supplementary Fig. 11 | Stability of the subclone detection process at distinguishing normal and malignant cells. In this sub-sampling analysis, 25%, 50%, and 75% of cells were first randomly removed at increments of 25% from the 12 AML patient samples (traces). Then, using increments of 5% from 75% to 95%, we studied breaking point of the clone detection procedure. After each removal of cells without replacement, scTherapy Step was rerun. The y-axis illustrates the percentage of correctly assigned cells before and after the cell removal. The expected curve is an anticipated percentage of correctly assigned cells from a beta binomial distribution and the shaded areas depict 95% quantile for each point estimate, providing confidence interval for the expected values under random cell assignment. Beta-binomial distribution was used to account for the varying proportion of malignant/healthy cell types due to removal of different cell percentages.

Supplementary References

1. Toi, M. *et al.* Adjuvant Abemaciclib Combined with Endocrine Therapy: Efficacy Results in monarchE Cohort 1. *The Oncologist* **28**, e77–e81 (2023).
2. Joensuu, H. *et al.* Adjuvant capecitabine in combination with docetaxel and cyclophosphamide plus epirubicin for breast cancer: an open-label, randomised controlled trial. *Lancet Oncol.* **10**, 1145–1151 (2009).
3. Hayes, D. F., Ethier, S. & Lippman, M. E. New guidelines for reporting of tumor marker studies in breast cancer research and treatment: REMARK. *Breast Cancer Res. Treat.* **100**, 237–238 (2006).
4. Nuzzo, F. *et al.* Effects on quality of life of weekly docetaxel-based chemotherapy in patients with locally advanced or metastatic breast cancer: results of a single-centre randomized phase 3 trial. *BMC Cancer* **11**, 75 (2011).
5. Knoop, A. S. *et al.* retrospective analysis of topoisomerase IIa amplifications and deletions as predictive markers in primary breast cancer patients randomly assigned to cyclophosphamide, methotrexate, and fluorouracil or cyclophosphamide, epirubicin, and fluorouracil: Danish Breast Cancer Cooperative Group. *J. Clin. Oncol. Off. J. Am. Soc. Clin. Oncol.* **23**, 7483–7490 (2005).
6. Martín, M. *et al.* Adjuvant docetaxel for high-risk, node-negative breast cancer. *N. Engl. J. Med.* **363**, 2200–2210 (2010).
7. Ek, L. *et al.* Randomized phase III trial of low-molecular-weight heparin enoxaparin in addition to standard treatment in small-cell lung cancer: the RASTEN trial. *Ann. Oncol. Off. J. Eur. Soc. Med. Oncol.* **29**, 398–404 (2018).
8. Bogart, J. *et al.* High-Dose Once-Daily Thoracic Radiotherapy in Limited-Stage Small-Cell Lung Cancer: CALGB 30610 (Alliance)/RTOG 0538. *J. Clin. Oncol. Off. J. Am. Soc. Clin. Oncol.* **41**, 2394–2402 (2023).
9. Niell, H. B. *et al.* Randomized phase III intergroup trial of etoposide and cisplatin with or without paclitaxel and granulocyte colony-stimulating factor in patients with extensive-stage small-cell lung cancer: Cancer and Leukemia Group B Trial 9732. *J. Clin. Oncol. Off. J. Am. Soc. Clin. Oncol.* **23**, 3752–3759 (2005).
10. Perry, M. C. *et al.* A phase III study of surgical resection and paclitaxel/carboplatin chemotherapy with or without adjuvant radiation therapy for resected stage III non-small-cell lung cancer: Cancer and Leukemia Group B 9734. *Clin. Lung Cancer* **8**, 268–272 (2007).
11. Efficace, F. *et al.* Is a patient's self-reported health-related quality of life a prognostic factor for survival in non-small-cell lung cancer patients? A multivariate analysis of prognostic factors of EORTC study 08975. *Ann. Oncol. Off. J. Eur. Soc. Med. Oncol.* **17**, 1698–1704 (2006).
12. Reck, M. *et al.* First-Line Nivolumab Plus Ipilimumab Versus Chemotherapy in Advanced NSCLC With 1% or Greater Tumor PD-L1 Expression: Patient-Reported Outcomes From CheckMate 227 Part 1. *J. Thorac. Oncol. Off. Publ. Int. Assoc. Study Lung Cancer* **16**, 665–676 (2021).
13. Langer, C. J. *et al.* Phase III trial comparing paclitaxel poliglumex (CT-2103, PPX) in combination with carboplatin versus standard paclitaxel and carboplatin in the treatment of PS 2 patients with chemotherapy-

naïve advanced non-small cell lung cancer. *J. Thorac. Oncol. Off. Publ. Int. Assoc. Study Lung Cancer* **3**, 623–630 (2008).
14. Pujol, J. L. *et al.* Etoposide plus cisplatin with or without the combination of 4'-epidoxorubicin plus cyclophosphamide in treatment of extensive small-cell lung cancer: a French Federation of Cancer Institutes multicenter phase III randomized study. *J. Natl. Cancer Inst.* **93**, 300–308 (2001).
15. Gridelli, C. *et al.* A randomised clinical trial of two docetaxel regimens (weekly vs 3 week) in the second-line treatment of non-small-cell lung cancer. The DISTAL 01 study. *Br. J. Cancer* **91**, 1996–2004 (2004).
16. Zhi, X. *et al.* VATS lobectomy facilitates the delivery of adjuvant docetaxel-carboplatin chemotherapy in patients with non-small cell lung cancer. *J. Thorac. Dis.* **5**, 578–584 (2013).
17. Scagliotti, G. *et al.* Phase III Multinational, Randomized, Double-Blind, Placebo-Controlled Study of Tivantinib (ARQ 197) Plus Erlotinib Versus Erlotinib Alone in Previously Treated Patients With Locally Advanced or Metastatic Nonsquamous Non-Small-Cell Lung Cancer. *J. Clin. Oncol. Off. J. Am. Soc. Clin. Oncol.* **33**, 2667–2674 (2015).
18. Appelbaum, F. R. *et al.* The clinical spectrum of adult acute myeloid leukaemia associated with core binding factor translocations. *Br. J. Haematol.* **135**, 165–173 (2006).
19. Zittoun, R. A. *et al.* Autologous or allogeneic bone marrow transplantation compared with intensive chemotherapy in acute myelogenous leukemia. European Organization for Research and Treatment of Cancer (EORTC) and the Gruppo Italiano Malattie Ematologiche Maligne dell'Adulto (GIMEMA) Leukemia Cooperative Groups. *N. Engl. J. Med.* **332**, 217–223 (1995).
20. Lin, Y.-H. *et al.* Global reduction of the epigenetic H3K79 methylation mark and increased chromosomal instability in CALM-AF10-positive leukemias. *Blood* **114**, 651–658 (2009).
21. Itzykson, R. *et al.* Impact of post-remission therapy in patients aged 65-70 years with de novo acute myeloid leukemia: a comparison of two concomitant randomized ALFA trials with overlapping age inclusion criteria. *Haematologica* **96**, 837–844 (2011).
22. Stone, R. M. *et al.* Phase III open-label randomized study of cytarabine in combination with amonafide L-malate or daunorubicin as induction therapy for patients with secondary acute myeloid leukemia. *J. Clin. Oncol. Off. J. Am. Soc. Clin. Oncol.* **33**, 1252–1257 (2015).
23. Muresan, B. *et al.* Comparing cure rates for gemtuzumab ozogamicin plus standard chemotherapy vs standard chemotherapy alone in acute myeloid leukemia patients. *Future Oncol. Lond. Engl.* **17**, 2883–2892 (2021).
24. Sarkozy, C. *et al.* Outcome of older patients with acute myeloid leukemia in first relapse. *Am. J. Hematol.* **88**, 758–764 (2013).
25. Lübbert, M. *et al.* 10-day decitabine versus 3 + 7 chemotherapy followed by allografting in older patients with acute myeloid leukaemia: an open-label, randomised, controlled, phase 3 trial. *Lancet Haematol.* **10**, e879–e889 (2023).
26. Amadori, S. *et al.* Sequential combination of gemtuzumab ozogamicin and standard chemotherapy in older patients with newly diagnosed acute myeloid leukemia: results of a randomized phase III trial by the EORTC and GIMEMA consortium (AML-17). *J. Clin. Oncol. Off. J. Am. Soc. Clin. Oncol.* **31**, 4424–4430 (2013).
27. Smith, C. C. *et al.* Molecular profile of FLT3-mutated relapsed/refractory patients with AML in the phase 3 ADMIRAL study of gilteritinib. *Blood Adv.* **6**, 2144–2155 (2022).
28. Harousseau, J.-L. *et al.* A randomized phase 3 study of tipifarnib compared with best supportive care, including hydroxyurea, in the treatment of newly diagnosed acute myeloid leukemia in patients 70 years or older. *Blood* **114**, 1166–1173 (2009).
29. Konstantinopoulos, P. A. *et al.* EPIK-O/ENGOT-OV61: alpelisib plus olaparib vs cytotoxic chemotherapy in high-grade serous ovarian cancer (phase III study). *Future Oncol. Lond. Engl.* **18**, 3481–3492 (2022).
30. Mariotta, M. *et al.* Dexamethasone-induced enhancement of resistance to ionizing radiation and chemotherapeutic agents in human tumor cells. *Strahlenther. Onkol. Organ Dtsch. Rontgengesellschaft Al* **175**, 392–396 (1999).
31. Möbus, V. *et al.* Phase III trial of high-dose sequential chemotherapy with peripheral blood stem cell support compared with standard dose chemotherapy for first-line treatment of advanced ovarian cancer: intergroup trial of the AGO-Ovar/AIO and EBMT. *J. Clin. Oncol. Off. J. Am. Soc. Clin. Oncol.* **25**, 4187–4193 (2007).
32. Moore, H. C. F. *et al.* Goserelin for ovarian protection during breast-cancer adjuvant chemotherapy. *N. Engl. J. Med.* **372**, 923–932 (2015).
33. Pujade-Lauraine, E. *et al.* Olaparib tablets as maintenance therapy in patients with platinum-sensitive, relapsed ovarian cancer and a BRCA1/2 mutation (SOLO2/ENGOT-Ov21): a double-blind, randomised, placebo-controlled, phase 3 trial. *Lancet Oncol.* **18**, 1274–1284 (2017).
34. Schettino, C. *et al.* Olaparib beyond progression compared with platinum chemotherapy after secondary cytoreductive surgery in patients with recurrent ovarian cancer: phase III randomized, open-label MITO 35b study, a project of the MITO-MANGO groups. *Int. J. Gynecol. Cancer Off. J. Int. Gynecol. Cancer Soc.* **32**, 799–803 (2022).
35. Gao, Q. *et al.* Olaparib Maintenance Monotherapy in Asian Patients with Platinum-Sensitive Relapsed Ovarian Cancer: Phase III Trial (L-MOCA). *Clin. Cancer Res. Off. J. Am. Assoc. Cancer Res.* **28**, 2278–2285 (2022).
36. DiSilvestro, P. *et al.* Overall Survival With Maintenance Olaparib at a 7-Year Follow-Up in Patients With Newly Diagnosed Advanced Ovarian Cancer and a BRCA Mutation: The SOLO1/GOG 3004 Trial. *J. Clin. Oncol. Off. J. Am. Soc. Clin. Oncol.* **41**, 609–617 (2023).
37. Penson, R. T. *et al.* Olaparib Versus Nonplatinum Chemotherapy in Patients With Platinum-Sensitive

Relapsed Ovarian Cancer and a Germline BRCA1/2 Mutation (SOLO3): A Randomized Phase III Trial. *J. Clin. Oncol. Off. J. Am. Soc. Clin. Oncol.* **38**, 1164–1174 (2020).
38. Ray-Coquard, I. *et al.* Olaparib plus Bevacizumab as First-Line Maintenance in Ovarian Cancer. *N. Engl. J. Med.* **381**, 2416–2428 (2019).
39. Pignata, S. *et al.* Carboplatin plus paclitaxel versus carboplatin plus pegylated liposomal doxorubicin as first-line treatment for patients with ovarian cancer: the MITO-2 randomized phase III trial. *J. Clin. Oncol. Off. J. Am. Soc. Clin. Oncol.* **29**, 3628–3635 (2011).
40. Pignata, S. *et al.* Carboplatin plus paclitaxel once a week versus every 3 weeks in patients with advanced ovarian cancer (MITO-7): a randomised, multicentre, open-label, phase 3 trial. *Lancet Oncol.* **15**, 396–405 (2014).
41. Parmar, M. K. B. *et al.* Paclitaxel plus platinum-based chemotherapy versus conventional platinum-based chemotherapy in women with relapsed ovarian cancer: the ICON4/AGO-OVAR-2.2 trial. *Lancet Lond. Engl.* **361**, 2099–2106 (2003).
42. Mannel, R. S. *et al.* A randomized phase III trial of IV carboplatin and paclitaxel × 3 courses followed by observation versus weekly maintenance low-dose paclitaxel in patients with early-stage ovarian carcinoma: a Gynecologic Oncology Group Study. *Gynecol. Oncol.* **122**, 89–94 (2011).
43. Spriggs, D. R. *et al.* Phase III randomized trial of intravenous cisplatin plus a 24- or 96-hour infusion of paclitaxel in epithelial ovarian cancer: a Gynecologic Oncology Group Study. *J. Clin. Oncol. Off. J. Am. Soc. Clin. Oncol.* **25**, 4466–4471 (2007).
44. Armstrong, D. K. *et al.* Intraperitoneal cisplatin and paclitaxel in ovarian cancer. *N. Engl. J. Med.* **354**, 34–43 (2006).
45. Marsh, S. *et al.* Pharmacogenetic assessment of toxicity and outcome after platinum plus taxane chemotherapy in ovarian cancer: the Scottish Randomised Trial in Ovarian Cancer. *J. Clin. Oncol. Off. J. Am. Soc. Clin. Oncol.* **25**, 4528–4535 (2007).
46. Coleman, R. L. *et al.* Veliparib with First-Line Chemotherapy and as Maintenance Therapy in Ovarian Cancer. *N. Engl. J. Med.* **381**, 2403–2415 (2019).
47. Pfisterer, J. *et al.* Randomized phase III trial of topotecan following carboplatin and paclitaxel in first-line treatment of advanced ovarian cancer: a gynecologic cancer intergroup trial of the AGO-OVAR and GINECO. *J. Natl. Cancer Inst.* **98**, 1036–1045 (2006).
48. Hoskins, P. *et al.* Advanced ovarian cancer: phase III randomized study of sequential cisplatin-topotecan and carboplatin-paclitaxel vs carboplatin-paclitaxel. *J. Natl. Cancer Inst.* **102**, 1547–1556 (2010).
49. Perren, T. J. *et al.* A phase 3 trial of bevacizumab in ovarian cancer. *N. Engl. J. Med.* **365**, 2484–2496 (2011).
50. Wenzel, L., Huang, H. Q., Monk, B. J., Rose, P. G. & Cella, D. Quality-of-life comparisons in a randomized trial of interval secondary cytoreduction in advanced ovarian carcinoma: a Gynecologic Oncology Group study. *J. Clin. Oncol. Off. J. Am. Soc. Clin. Oncol.* **23**, 5605–5612 (2005).
51. Hewitt, D. B. *et al.* A Phase 3 Randomized Clinical Trial of Chemotherapy With or Without Algenpantucel-L (HyperAcute-Pancreas) Immunotherapy in Subjects With Borderline Resectable or Locally Advanced Unresectable Pancreatic Cancer. *Ann. Surg.* **275**, 45–53 (2022).
52. Conroy, T. *et al.* FOLFIRINOX versus gemcitabine for metastatic pancreatic cancer. *N. Engl. J. Med.* **364**, 1817–1825 (2011).
53. Reni, M. *et al.* A randomised phase 2 trial of nab-paclitaxel plus gemcitabine with or without capecitabine and cisplatin in locally advanced or borderline resectable pancreatic adenocarcinoma. *Eur. J. Cancer Oxf. Engl. 1990* **102**, 95–102 (2018).
54. Huh, G. *et al.* Gemcitabine plus Nab-paclitaxel as a second-line treatment following FOLFIRINOX failure in advanced pancreatic cancer: a multicenter, single-arm, open-label, phase 2 trial. *Ther. Adv. Med. Oncol.* **13**, 17588359211056179 (2021).
