# Supplementary material for: Single-cell transcriptomes identify patient-tailored therapies for selective co-inhibition of cancer clones
Source: Nat Commun. 2024 Oct 3;15:8579. doi: 10.1038/s41467-024-52980-5 (PMC11450203; doi:10.1038/s41467-024-52980-5)
Supplement: Supplementary file 3 — Reporting Summary [file 41467_2024_52980_MOESM3_ESM.pdf]

nature portfolio

Corresponding author(s): Tero Aittokallio

Last updated by author(s): Sep 26, 2024

Reporting Summary

Nature Portfolio wishes to improve the reproducibility of the work that we publish. This form provides structure for consistency and transparency in reporting. For further information on Nature Portfolio policies, see our Editorial Policies and the Editorial Policy Checklist.

Statistics

For all statistical analyses, confirm that the following items are present in the figure legend, table legend, main text, or Methods section.

n/a	Confirmed	
☐	☒	The exact sample size (*n*) for each experimental group/condition, given as a discrete number and unit of measurement
☐	☒	A statement on whether measurements were taken from distinct samples or whether the same sample was measured repeatedly
☐	☒	The statistical test(s) used AND whether they are one- or two-sided<br>*Only common tests should be described solely by name; describe more complex techniques in the Methods section.*
☐	☒	A description of all covariates tested
☐	☒	A description of any assumptions or corrections, such as tests of normality and adjustment for multiple comparisons
☐	☒	A full description of the statistical parameters including central tendency (e.g. means) or other basic estimates (e.g. regression coefficient) AND variation (e.g. standard deviation) or associated estimates of uncertainty (e.g. confidence intervals)
☐	☒	For null hypothesis testing, the test statistic (e.g. *F*, *t*, *r*) with confidence intervals, effect sizes, degrees of freedom and *P* value noted<br>*Give P values as exact values whenever suitable.*
☒	☐	For Bayesian analysis, information on the choice of priors and Markov chain Monte Carlo settings
☒	☐	For hierarchical and complex designs, identification of the appropriate level for tests and full reporting of outcomes
☒	☐	Estimates of effect sizes (e.g. Cohen's *d*, Pearson's *r*), indicating how they were calculated

Our web collection on statistics for biologists contains articles on many of the points above.

Software and code

Policy information about availability of computer code

Data collection	PharmacoGx R-package v3.4.0 for collecting drug-induced viability data from multiple cancer cell line screening studies
Data analysis	The R codes for reproducing the results and making new patient-specific predictions are available on GitHub (https://github.com/kris-nader/scTherapy) and Zenodo (https://doi.org/10.5281/zenodo.13340796). Docker image that encapsulates all the relevant dependencies and ensures compatibility across different environments is available on Docker Hub (https://hub.docker.com/r/kmnader/sctherapy). Separate docker image compatible with the latest Seurat version v5 has also been available (https://hub.docker.com/r/kmnader/sctherapy_v5).<br><br>R-packages: sctype v1.0.0, infercnv v1.3.3, biomart v2.56.1, Seurat v4.3.0, SeuratObject v4.1.3, HGNChelper v0.8.1, copykat v1.1.0, SCEVAN v1.0.1, Seurat v5.0.2 , SeuratObject v5.0.1. All scripts and functions have been made compatible with versions 4 and 5 of Seurat and SeuratObjects. Two Docker images are available using all base packages with Seurat v4.3.0 and Seurat v5.0.2

For manuscripts utilizing custom algorithms or software that are central to the research but not yet described in published literature, software must be made available to editors and reviewers. We strongly encourage code deposition in a community repository (e.g. GitHub). See the Nature Portfolio guidelines for submitting code & software for further information.

Data

Policy information about availability of data

All manuscripts must include a data availability statement. This statement should provide the following information, where applicable:

- Accession codes, unique identifiers, or web links for publicly available datasets
- A description of any restrictions on data availability
- For clinical datasets or third party data, please ensure that the statement adheres to our policy

The previously published single-cell RNA sequencing data for 9 AML patients are available in the European Genome-Phenome Archive (EGA) under accession codes: EGAS0000100461433 (AML patients 2, 3, 8 and 10) and EGAS0000100444469 (AML patients 1, 4, 7, 8, and 11). The single-cell RNA sequencing data generated in this study for AML patients 5, 6, and 12 are available through Sequence Read Archive (SRA accession numbers; Patient 5: SRR30720408- https://www.ncbi.nlm.nih.gov/sra/?term=SRR30720408; Patient 6: SRR30720407- https://www.ncbi.nlm.nih.gov/sra/?term=SRR30720407: Patient 12: SRR30720406- https://www.ncbi.nlm.nih.gov/sra/?term=SRR30720406). The processed Seurat objects were deposited to Zenodo (https://doi.org/10.5281/zenodo.13340927)71. The previously published single-cell RNA sequencing data for the 5 HGSC patient samples are available on EGA under accession codes: EGAS0000100501025 (HGSC patients 1, 2, 3 and 4) and EGAS0000100506626 (HGSC patient 5). The publicly available data used in this study are accessible in the Connectivity Map LINCS 2020 (https://clue.io/data)22, PharmacoDB (https://pharmacodb.ca), and PubChem Compound database (https://www.ncbi.nlm.nih.gov/pccompound). The scRNA-seq data from cancer patients in other tumor types were obtained from the dataset curated by Gavish et al (https://www.weizmann.ac.il/sites/3CA). The source data generated in this study are provided in the Supplementary Information or Source Data file. The remaining data are available within the Article, Supplementary Information or Source Data file.

Research involving human participants, their data, or biological material

Policy information about studies with human participants or human data. See also policy information about sex, gender (identity/presentation), and sexual orientation and race, ethnicity and racism.

Reporting on sex and gender	In the study cohort, a total of 12 (7 patients with AML and 5 HGSC) participants assigned female at birth biologically, and 5 participants (patients with AML) assigned male at birth biologically. The sex of AML patients is reported in Suppl. Table 1. All the ovarian cancer patients were female at birth biologically.
Reporting on race, ethnicity, or other socially relevant groupings	This is a n=1 personalized medicine study, i.e., all the model predictions and experimental testing were done for each patient separately, using their scRNA-seq data and single-cell drug assays, respectively. Therefore, age, sex, gender, race, ethnicity, or other social parameters were not considered as confounding factors.
Population characteristics	The patient characteristics are reported in Suppl. Tables 1-3. See also above for the patient-specific analyses. This is not a population-level association analysis, instead a personalized prediction of multi-targeting drugs for each patient separately.
Recruitment	Patients were recruited as part of two ongoing translational studies for AML and HGSC. No compensation was provided for the participants. The patient samples for this study were selected based on the availability of required data for modeling (scRNA-seq) and primary cells for experimental validation (single-cell drug assays). These selection criteria are not expected to bias the results, since the selection was not based on any patient characteristics, such as gender, or clinical outcomes.
Ethics oversight	This research complies with all relevant ethical regulations, approved by the institutional review boards, who approved the use of the human samples in the study. The AML patient samples and data were collected and published with signed informed consent in accordance with the Declaration of Helsinki (HUS Ethical Committee Statement 303/13/03/01/2011, latest amendment 7 dated June 15, 2016. Latest HUS study permit HUS/395/2018 dated February 13, 2018)). The HGSC patient samples were collected as a part of a larger study cohort, where all patients participating in the study provided written informed consent. The study and the use of all clinical material have been approved by The Ethics Committee of the Hospital District of Southwest Finland (ETMK) under decision number EMTK: 145/1801/2015.

Note that full information on the approval of the study protocol must also be provided in the manuscript.

Field-specific reporting

Please select the one below that is the best fit for your research. If you are not sure, read the appropriate sections before making your selection.

[x] Life sciences [ ] Behavioural & social sciences [ ] Ecological, evolutionary & environmental sciences

For a reference copy of the document with all sections, see nature.com/documents/nr-reporting-summary-flat.pdf

Life sciences study design

All studies must disclose on these points even when the disclosure is negative.

Sample size	The samples for this study were selected based on the availability of required data for modelling (scRNA-seq) and primary cells for experimental validation (single-cell drug assays). Since this is a n=1 precision oncology approach (i.e., the predictions and experimental testing is carried out for each patient separately), no statistical method was used to predetermine sample size. The cohort size does not determine the significance of the results. Instead, the model predictions were validated in multiple (n>10) treatment experiments in the patient cells, and statistical analyses of the experimental validations showed that the prediction accuracies are significant, and therefore the number of

	drug treatments is sufficient in each patient. Since we make patient-specific predictions and validations, instead of testing cohort-level statistical associations, statistical power analysis is not so relevant here (i.e., the focus is on predictive power, instead of statistical power).
Data exclusions	No data exclusion.
Replication	All the validation drug assays were replicated either 2 or 3 times, depending on the availability of primary patient cells. All the replicate measurements were successful in the sense that the standard deviations were within an expected range based on previous studies.
Randomization	Randomization is not relevant, since this is not a case-control study, rather the non-cancerous cells from each patient sample were separately used as control for the particular patient's cancer cell responses.
Blinding	Blinding is not relevant, since this is not a case-control study. The experimental validation of the model predictions was made after the predictions. Experimental researchers were blinded to the predictions.

Reporting for specific materials, systems and methods

We require information from authors about some types of materials, experimental systems and methods used in many studies. Here, indicate whether each material, system or method listed is relevant to your study. If you are not sure if a list item applies to your research, read the appropriate section before selecting a response.

Materials & experimental systems

n/a	Involved in the study	
☐	☒	Antibodies
☒	☐	Eukaryotic cell lines
☒	☐	Palaeontology and archaeology
☒	☐	Animals and other organisms
☒	☐	Clinical data
☒	☐	Dual use research of concern
☒	☐	Plants

Methods

n/a	Involved in the study	
☒	☐	ChIP-seq
☒	☐	Flow cytometry
☒	☐	MRI-based neuroimaging

Antibodies

Antibodies used

Anti-PAX8 rabbit polyclonal antibody (dilution 1:100), IgG, Proteintech, #10336-1-AP; anti-EpCam mouse monoclonal (dilution 1:500), IgG1, Santa Cruz, #sc-25308 (C-10); anti-GAPDH mouse monoclonal (dilution 1:5000), IgM, Novus, #NB300-221 (1D4); BV785 Mouse Anti-Human CD14 (dilution 1:200)(Cat. no. 367141, Biolegend); VB515 Recombinant Anti-Human CD56 (dilution 1:400)(Cat. no. 130-114-743, Miltenyi); V500 Mouse Anti-Human CD45 (dilution 1:240)(Cat. no. 560777, BD Biosciences); BV650 Mouse Anti-Human CD19 (dilution 1:120)(Cat. no. 563227, BD Biosciences); PE-Cy7 Mouse Anti-Human CD3 (dilution 1:150)(Cat. no. 560910, BD Biosciences); PE Mouse Anti-Human CD34 (dilution 1:240)(Cat. no. 550761, BD Biosciences); BV421 Mouse Anti-Human CD38 (dilution 1:600)(Cat. no. 562444, BD Biosciences); APC Mouse Anti-Human CD117 (dilution 1:600)(Cat. no. 567127, BD Biosciences); APC-Fire 750 Annexin V (dilution 1:80)(Cat. no. 640952, Biolegend).

Validation

A. The antibodies used in the ovarian cancer profiling share a long-standing published track record of application-specific performance. Antigen specificity in immunofluorescence (IF) and Western blotting (WB) has been validated by manufacturers and/or confirmed in-house or in studies by others.

Anti-PAX8 rabbit polyclonal antibody, IgG, Proteintech, #10336-1-AP was used in IF and WB. The antibody has received extensive validation: 106 articles support its specificity in IF, and 55 articles report specificity in WB (https://www.ptglab.com/products/PAX8-Antibody-10336-1-AP.htm), and 502 references to the antibody can be found in the Antibodypedia database (https://www.antibodypedia.com/gene/9626/PAX8). The vendor states that the antibody is validated on the PAX8 knock-down and knock-out models; 5 articles reference the statement. We tested the #10336-1-AP antibody's performance in in-house IF and WB protocols using conventional cancer cell lines before the experiments on ovarian cancer organoids. For IF, we confirmed high nuclear expression of HGSC-specific transcription factor PAX8 in HGSC cell line OVCAR3 (positive control) and detected the lack of PAX8 in NSCLC cell line A549 (negative control). For WB, we confirmed immunodetection of PAX8 bands (near 55 kDa) in HGSC cell lines KURAMOCHI and OVCAR4 (positive controls). According to Proteintech, the antibody detects bands between 48 and 58 kDa, reacting with 5 isoforms of PAX8. The bands were absent in lysates of colon cancer cell line RKO and in immortalized retinal epithelium cell line RPE (negative controls), confirming the reagent specificity.

Anti-EpCam mouse monoclonal, IgG1, Santa Cruz, #sc-25308 (C-10) has 13 records in the Labome Validated Antibody Database https://www.labome.com/product/Santa-Cruz-Biotechnology/sc-25308.html and 75 entries in CiteAb database (https://www.citeab.com/antibodies/791613-sc-25308-ep-cam-antibody-c-10?des=bad7807fa3734a5a), including the references to EpCam band detection on WB. Validating the sc-25308 antibody specificity, articles by Sankpal et al. 2011 (https://link.springer.com/article/10.1186/bcr3070#Sec2) and Banyard et al., 2014 (https://link.springer.com/article/10.1186/1471-2407-14-387#Sec2) demonstrate the lack of band detection in EpCam RNAi-depleted MDA-MB-231, Ca1, and DU-145 cancer cell lines. According to the manufacturer, the sc-25308 antibody is suitable as a control antibody for EpCAM siRNA. Optimal antibody performance in our WB experiments was justified by detecting the band corresponding to the predicted size (40-43 kDa) in ovarian cancer cells and the lack of the band in the stromal cell samples.

Anti-GAPDH mouse monoclonal, IgM, Novus, #NB300-221 (1D4) is a validated antibody, as recorded in the Labome Validated

Antibody Database https://www.labome.com/product/Novus-Biologicals/NB300-221.html. Lack of GAPDH protein band on GAPDH RNAi-depleted samples confirms NB300-221 antibody specificity (Grolla et al., 2020 (https://www.ncbi.nlm.nih.gov/pmc/articles/PMC7076215/). Optimal antibody performance in our WB experiments was justified by detecting the band corresponding to the predicted size (37 kDa). NB300-221 antibody has over 300 cited applications in WB listed by the manufacturer https://www.novusbio.com/products/gapdh-antibody-1d4_nb300-221?utm_source=antibodypedia&utm_medium=referral&utm_campaign=product#PublicationSection and in Antibodypedia (https://www.antibodypedia.com/gene/3923/GAPDH/antibody/27538/NB300-221).

B. All the antibodies used for the flow cytometry application have been validated before. Reactivity to humans has been controlled by manufacturer QC statements. Below are the Manufacturer's citations together with catalog numbers for the flow cytometry primary antibodies.

BV785 Mouse Anti-Human CD14 (Cat. no. 367141, Biolegend)
1. Fridlender ZG, et al. 1999. Hum. Immunol. 11:1028.
2. Devitt A, et al. 1998. Nature 6675:505.

VB515 Recombinant Anti-Human CD56 (Cat. no. 130-114-743, Miltenyi)
1. Popkin, D. L. et al. (2018) In vitro evidence that combination therapy with CD16-bearing NK-92 cells and FDA-approved Alefacept can selectively target the latent HIV reservoir in CD4+ CD2hi memory T cells. Front Immunol 9: 2552
2. Soldierer, M. et al. (2022) Genetic Engineering and Enrichment of Human NK Cells for CAR-Enhanced Immunotherapy of Hematological Malignancies, Front Immunol 13: doi: 10.3389/fimmu.2022.847008
3. Boin, F. et al. (2017) Flow cytometric discrimination of seven lineage markers by using two fluorochromes. PLoS One 12 (11)
4. Canté-Barrett, K. et al. (2017) Loss of CD44dim expression from early progenitor cells marks T-cell lineage commitment in the human thymus. Front Immunol 8: 32
5. Croci, S. et al. (2018) Higher frequencies of lymphocytes expressing the natural killer group 2D receptor in patients with Behçet disease. Front Immunol 9: 2157
6. Vivier, E. et al. (2018) Anti-NKG2A mAb Is a Checkpoint Inhibitor that Promotes Anti-tumor Immunity by Unleashing Both T and NK Cells. Cell 175 (7): 1731-1743
7. Camp, B. V. et al. (1990) Plasma cells in multiple myeloma express a natural killer cell- associated antigen: CD56 (NKH-1; Leu-19). Blood 76: 377-382
8. Grant, M. D. et al. (2018) Natural Killer Cells Adapt to Cytomegalovirus Along a Functionally Static Phenotypic Spectrum in Human Immunodeficiency Virus Infection. Front Immunol 9: 2494
9. Petty, R. D. et al. (2016) MicroRNAs hsa-miR-99b, hsa-miR-330, hsa-miR-126 and hsa-miR-30c: potential diagnostic biomarkers in natural killer (NK) cells of patients with chronic fatigue syndrome (CFS)/ myalgic encephalomyelitis (ME). PLoS One 11: e0150904
10. Delso-Vallejo, M. et al. (2017) Influence of irradiated peripheral blood mononuclear cells on both ex vivo proliferation of human natural killer cells and change in cellular property. Front Immunol 8
11. Veluchamy, J. et al. (2017) In vivo efficacy of umbilical cord blood stem cell-derived NK cells in the treatment of metastatic colorectal cancer. Front Immunol 8: 87
12. Dong, P. et al. (2017) Simultaneous detection of decidual Th1/Th2 and NK1/NK2 immunophenotyping in unknown recurrent miscarriage using 8-color flow cytometry with FSC/Vt extended strategy. Biosci. Rep. 37 (3)

V500 Mouse Anti-Human CD45 (Cat. no. 560777, BD Biosciences)
1. Bradstock KF, Janossy G, Pizzolo G, et al. Subpopulations of normal and leukemic human thymocytes: an analysis with the use of monoclonal antibodies. J Natl Cancer Inst. 1980; 65(1):33-42. (Biology).
2. Hermiston ML, Xu Z, Weiss A. CD45: a critical regulator of signaling thresholds in immune cells. Annu Rev Immunol. 2003; 21:107-137. (Biology).
3. Knapp W. W. Knapp .. et al., ed. Leucocyte typing IV : white cell differentiation antigens. Oxford New York: Oxford University Press; 1989:1-1182.
4. Loken MR, Brosnan JM, Bach BA, Ault KA. Establishing optimal lymphocyte gates for immunophenotyping by flow cytometry. Cytometry. 1990; 11(4):453-459. (Biology).
5. Terry LA, Brown MH, Beverley PC. The monoclonal antibody, UCHL1, recognizes a 180,000 MW component of the human leucocyte-common antigen, CD45. Immunology. 1988; 64(2):331-336. (Biology).
6. Terstappen LW, Levin J. Bone marrow cell differential counts obtained by multidimensional flow cytometry. Blood Cells. 1992; 18 (2):311-330. (Biology).
7. Trowbridge IS, Thomas ML. CD45: an emerging role as a protein tyrosine phosphatase required for lymphocyte activation and development. Annu Rev Immunol. 1994; 12:85-116. (Biology).
8. Zola H. Leukocyte and stromal cell molecules : the CD markers. Hoboken, N.J.: Wiley-Liss; 2007.

BV650 Mouse Anti-Human CD19 (Cat. no. 563227, BD Biosciences)
1. Dörken B, Möller P, Pezzutto A, Schwartz-Albiez R, Moldenhauer G. B-cell antigens: CD19. In: Knapp W. W. Knapp .. et al., ed. Leucocyte typing IV : white cell differentiation antigens. Oxford New York: Oxford University Press; 1989:34-36.
2. Loken MR, Shah VO, Dattilio KL, Civin CI. Flow cytometric analysis of human bone marrow. II. Normal B lymphocyte development. Blood. 1987; 70(5):1316-1324. (Biology).
3. Moldenhauer G, Dörken B, Schwartz R, Pezzutto A, Knops J, Hammerling GJ. Analysis of ten B lymphocyte-specific workshop monoclonal antibodies. In: Reinherz EL, Haynes BF, Nadler LM, Bernstein ID, ed. Leukocyte Typing II: Human B Lymphocytes. New York: Springer-Verlag; 1986:61-67.
4. Nadler LM, Anderson KC, Marti G, et al. B4, a human B lymphocyte-associated antigen expressed on normal, mitogen-activated, and malignant B lymphocytes. J Immunol. 1983; 131(1):244-250. (Clone-specific: Flow cytometry).
5. Nadler LM. B Cell/Leukemia Panel Workshop: summary and comments. In: Reinherz EL, Haynes BF, Nadler LM, Bernstein ID, ed. Leukocyte Typing II: Human B Lymphocytes. New York: Springer-Verlag; 1986:3-43.
6. Reichert T, DeBruyere M, Deneys V, et al. Lymphocyte subset reference ranges in adult Caucasians. Clin Immunol Immunopathol. 1991; 60(2):190-208. (Biology).

7. Tedder TF, Zhou LJ, Engel P. The CD19/CD21 signal transduction complex of B lymphocytes. Immunol Today. 1994; 15(9):437-442. (Biology).

PE-Cy7 Mouse Anti-Human CD3 (Cat. no. 560910, BD Biosciences)
1. Barclay NA, Brown MH, Birkeland ML, et al, ed. The Leukocyte Antigen FactsBook. San Diego, CA: Academic Press; 1997.
2. Beverley PC, Callard RE. Distinctive functional characteristics of human "T" lymphocytes defined by E rosetting or a monoclonal anti-T cell antibody. Eur J Immunol. 1981; 11(4):329-334. (Biology).
3. Knapp W. W. Knapp .. et al., ed. Leucocyte typing IV : white cell differentiation antigens. Oxford New York: Oxford University Press; 1989:1-1182.
4. Lanier LL, Allison JP, Phillips JH. Correlation of cell surface antigen expression on human thymocytes by multi-color flow cytometric analysis: implications for differentiation. J Immunol. 1986; 137(8):2501-2507. (Biology).
5. McMichael AJ. A.J. McMichael .. et al., ed. Leucocyte typing III : white cell differentiation antigens. Oxford New York: Oxford University Press; 1987:1-1050.
6. Roederer M, Kantor AB, Parks DR, Herzenberg LA. Cy7PE and Cy7APC: bright new probes for immunofluorescence. Cytometry. 1996; 24(3):191-197. (Biology).
7. Schlossman SF. Stuart F. Schlossman .. et al., ed. Leucocyte typing V : white cell differentiation antigens : proceedings of the fifth international workshop and conference held in Boston, USA, 3-7 November, 1993. Oxford: Oxford University Press; 1995.

PE Mouse Anti-Human CD34 (Cat. no. 550761, BD Biosciences)
1. Egeland T, Tjonnfjord G, Steen R, Gaudernack G, Thorsby E. Positive selection of bone marrow-derived CD34 positive cells for possible stem cell transplantation. Transplant Proc. 1993; 25(1):1261-1263. (Biology).
2. Kishimoto T. Tadamitsu Kishimoto .. et al., ed. Leucocyte typing VI : white cell differentiation antigens : proceedings of the sixth international workshop and conference held in Kobe, Japan, 10-14 November 1996. New York: Garland Pub.; 1997.
3. Knapp W. W. Knapp .. et al., ed. Leucocyte typing IV : white cell differentiation antigens. Oxford New York: Oxford University Press; 1989:1-1182.
4. Nishio H, Tada J, Hashiyama N, Hirn J, Ingles-Esteven J, Suda T. CD34. 1999. Available: http://mpr.nci.nih.gov/prow/guide/968267813_g.htm 2006, February 8.
5. Owens MA, Loken MR. Peripheral blood stem cell quantitation. In: Owens MA, Loken MR. Flow Cytometry Principles for Clinical Laboratory Practice. New York: John Wiley & Sons; 1995:128.
6. Schlossman SF. Stuart F. Schlossman .. et al., ed. Leucocyte typing V : white cell differentiation antigens : proceedings of the fifth international workshop and conference held in Boston, USA, 3-7 November, 1993. Oxford: Oxford University Press; 1995.

BV421 Mouse Anti-Human CD38 (Cat. no. 562444, BD Biosciences)
1. McMichael AJ. A.J. McMichael .. et al., ed. Leucocyte typing III : white cell differentiation antigens. Oxford New York: Oxford University Press; 1987:1-1050.
2. Schlossman SF. Stuart F. Schlossman .. et al., ed. Leucocyte typing V : white cell differentiation antigens : proceedings of the fifth international workshop and conference held in Boston, USA, 3-7 November, 1993. Oxford: Oxford University Press; 1995.

APC Mouse Anti-Human CD117 (Cat. no. 567127, BD Biosciences)
1. Ashman LK, Buhring HJ, Aylett GW, Broudy VC, Muller C. Epitope mapping and functional studies with three monoclonal antibodies to the c-kit receptor tyrosine kinase, YB5.B8, 17F11, and SR-1. J Cell Physiol. 1994; 158(3):545-554. (Biology: Flow cytometry).
2. Ashman LK, Cambareri A, Nguyen L, Bühring H-J. CD117 workshop panel report. In: Kishimoto T. Tadamitsu Kishimoto .. et al., ed. Leucocyte typing VI : white cell differentiation antigens : proceedings of the sixth international workshop and conference held in Kobe, Japan, 10-14 November 1996. New York: Garland Pub.; 1997:816-818.
3. Rappold I, Ziegler BL, Kohler I, et al. Functional and phenotypic characterization of cord blood and bone marrow subsets expressing FLT3 (CD135) receptor tyrosine kinase. Blood. 1997; 90(1):111-125. (Immunogen).

APC-Fire 750 Annexin V (Cat. no. 640952, Biolegend)
1. de Winde CM, et al. 2021. iScience. 24:102976.
2. Parayath NN, et al. 2020. Nat Commun. 4.680555556.
